# Re: Comparison of Gleason upgrading rates in transrectal ultrasound systematic random biopsies versus US-MRI fusion biopsies for prostate cancer

**DOI:** 10.1590/S1677-5538.IBJU.2019.0048

**Published:** 2019-07-27

**Authors:** Ibrahim Halil Bozkurt, Ertugrul Sefik, Ismail Basmaci, Serdar Celik

**Affiliations:** 1 HSU Izmir Bozyaka Training and Research Hospital Urology Clinic, Izmir, Turkey

*To the editor,*

We have read the paper, “Comparison of Gleason upgrading rates in transrectal ultrasound systematic random biopsies versus US-MRI fusion biopsies for prostate cancer” with great interest and appreciate the work of the authors (1). They have compared the Gleason upgrading (GU) rates and tried to determine the concordance of the Gleason scores in the biopsy versus final pathology after surgery in patients who underwent transrectal ultrasound (TRUS) systematic random biopsies (SRB) versus US-MRI FB for prostate cancer (PCa).

They found that the GU rate was higher in TRUS SRB group (31.5% vs. 16.4%; p=0.027). According to the Gleason grade pattern, GU was higher in TRUS SRB group compared to US-MRI FB group (40.4% vs. 23.3%; p=0.020).

Authors concluded that US-MRI FB appears to be related to a decrease in GU rate and an increase in concordance between biopsy and final pathology compared to TRUS SRB, suggesting that performing US-MRI FB leads to greater accuracy of diagnosis and better treatment decisions.

We have a few queries: we know from the previous studies that around 10% of the tumors were undetected by MRI and additional tumor foci may be detected in the histological examination of the final pathological specimen (2). In the recent paper it was stated that a decrease in GU rate was detected with the use of US-MRI FB. We kindly ask the authors if they detected any additional tumor foci in the radical prostatectomy specimen of US-MRI FB group or the GU just belongs to the tumor detected via fusion biopsy.

In the recent study eight patients that have Gleason score ≤6 on US-MRI FB undergone radical prostatectomy. It is known that active surveillance is one of the best treatment options for very low risk prostate cancer. The number of the positive cores and percentage of the each fragment/core involved are the important parameters to decide active surveillance in the patients with T1c and Gleason score ≤6 /grade group 1 and PSA<10 ng/mL. Concerning the fact that limited biopsy cores were obtained in the MRI-targeted biopsy group; we kindly ask the authors which criteria they used for the selection of patients to active surveillance. In the era of MRI-targeted biopsy do the authors propose alternative criteria for selection of patients to active surveillance?

When speaking about the concordance of US-MRI FB and final pathology Gleason score another important issue is down grading of Gleason score. What was the down grading in the US-MRI FB group and was it different from TRUS SRB group in this study?
